# Spermine: A prospective treatment for high glucose-induced myocardial fibrosis in db/db mice

**DOI:** 10.22038/ijbms.2025.83464.18060

**Published:** 2025

**Authors:** Yong Liu, Xinyu Liu, Shuang Li, Tianshuang Xu, Yang Lu, Tao Wang, Hui Yuan

**Affiliations:** 1 Animal Research Institute, Research Department, Mudanjiang Medical University, Mudanjiang, China; 2 School of Basic Medical Sciences, Mudanjiang Medical University, Mudanjiang, China; 3 Department of Immunology, Mudanjiang Medical University, Heilongjiang, China; 4 Mudanjiang Traditional Chinese Medicine Hospital, Mudanjiang, China; 5 School of Stomatology, Mudanjiang Medical University, Mudanjiang, China

**Keywords:** Cardiac fibroblast, Myocardial fibrosis, Spermine, TGF-β1/Smads, Type 2 diabetes

## Abstract

**Objective(s):**

This study explored the molecular mechanism by which exogenous spermine attenuates diabetic cardiomyopathy (DCM)-induced myocardial fibrosis.

**Materials and Methods:**

db/db mice and primary neonatal mouse cardiac fibroblasts were used to conduct *in vivo *and *in vitro* experiments. The levels of total cholesterol (TC), triglycerides (TG), creatine kinase isoenzyme (CK-MB), troponin I (cTnI), and lactate dehydrogenase (LDH) were measured. Heart function and collagen deposition were assessed using echocardiographic analysis, Masson staining, and Sirius red staining. Cell proliferation and migration were analyzed using EdU and transwell assays. Relevant protein expression was evaluated by immunohistochemistry and western blot.

**Results:**

After 12 weeks, the mice in the type 2 diabetes (T2D) group exhibited increased blood glucose, TG, TC, and serum myocardial marker enzyme levels. Ejection fraction (EF) and left ventricular fractional shortening left ventricular fractional shortening (FS) decreased, while LVIDs and LVIDd increased. Significant collagen fiber deposition and increased HW/TL ratio, SSAT, α-SMA, TGF-β1, and Collagen-I/III expression was observed in myocardial tissue. Conversely, ODC expression was down-regulated. In the T2D + spermine (SP) group, these trends were reversed. *In vitro*, high glucose conditions led to increased proliferation of cardiac fibroblasts. SSAT, α-SMA, TGF-β1, Collagen-I/III, MMP-2, MMP-9, p-Smad-2, TβRI, and TβRII were up-regulated, while ornithine decarboxylase (ODC) expression was down-regulated. Interestingly, these changes were reversed in the HG + SP group.

**Conclusion:**

Our findings demonstrate that SP reduces collagen synthesis and secretion by inhibiting the TGF-β1/Smads signaling pathway. These results provide new insights into potential therapeutic approaches for DCM.

## Introduction

Diabetic cardiomyopathy (DCM) is a heart disease that occurs exclusively in individuals with diabetes. It is distinct from hypertensive heart disease, coronary atherosclerotic heart disease, and other cardiomyopathies caused by heart-related conditions(1, 2). In the progression of DCM, severe metabolic abnormalities and notable microangiopathies develop in the myocardial tissue, which can lead to diffuse necrosis. Subclinical cardiac dysfunctions such as ventricular stiffness, myocardial hypertrophy, and myocardial fibrosis may also arise. In the early stages of DCM, diastolic function is significantly impaired. At the same time, severe systolic dysfunction typically develops in later stages, eventually leading to complications such as arrhythmia, heart failure, shock, and even sudden cardiac death (3-5). 

Polyamines are small, linear, amino acid-derived branched cationic molecules widely distributed in prokaryotic and eukaryotic cells. These molecules, which include spermine and putrescine, are involved in various cellular biological processes (6). They regulate DNA synthesis, cell cycle progression, cell proliferation and differentiation, aging, endoplasmic reticulum (ER) stress, oxidative stress, and ion channel activity. Moreover, they exhibit anti-inflammatory, anti-oxidant, and anti-free radical effects (7, 8). Previous studies have demonstrated that polyamine metabolic disturbances are present in experimental animal models of myocardial ischemia-reperfusion injury in rats, and administration of exogenous spermine has been shown to provide adequate protective effects (9). However, there has been limited research on the physiological effects and underlying mechanisms of spermine in the context of DCM. 

In this study, we combined animal and cell experiments to investigate the molecular mechanisms by which exogenous spermine alleviates diabetic myocardial fibrosis. Our findings aim to provide an experimental foundation and identify new therapeutic targets for the effective prevention and treatment of DCM.

## Materials and Methods

### Drugs and reagents

Spermine was purchased from Sigma; The CK-MB test kit was purchased from Solebault; cTnI test kit was purchased from Jiubang Biology; Insulin test kit was purchased from Lyle Bio; SSAT, α-SMA, TGF-β_1_, Collagen-I/III, MMP-2, MMP-9, p-smad-2, TβRI and TβRII (abclonal, Wuhan, China); Anti-rat/Rabbit IgG Antibody 1:5000 dilution (LBI0311, LABEST, Beijing, China); BCA protein detection kit (P0009, Beyotime, Shanghai, China).

### Experimental animal model

C57BL/6 mice and db/db mice (Male, weight 20 ± 2 g) were used, purchased from GemPharmatech Co. Ltd. db/db mice are a widely used model for studying type 2 diabetes. These mice have a genetic background based on the C57BL/6 strain, with a mutation in the leptin receptor gene (Lepr). This mutation leads to severe insulin resistance; even insulin interventions fail to control hyperglycemia in db/db mice effectively. They spontaneously develop type 2 diabetes due to the leptin receptor defect, making them a valuable model for studying endocrine disorders, neurological diseases, and cardiac complications associated with abnormal glucose and lipid metabolism(10, 11). They were divided into three groups: normal Control group (Control group), type 2 diabetes group (T2D group), and type 2 diabetes + spermine treatment group (T2D+SP group). db/db mice aged 7 weeks were selected as type 2 diabetes model animals after adaptive feeding under SPF conditions for 1 week after random blood glucose ≥ 16.7 mmol/l. The T2D+SP group was treated with 4 mg/kg intraperitoneal injection of spermine every other day (prepared with 0.9 % NaCl solution).

### Cell culture

Primary myocardial fibroblasts were isolated from C57BL/6 mice within 1–3 days of birth (purchased from the Experimental Animal Center of Mudanjiang Medical University). They were divided into five groups: Control group: DMEM culture medium with final concentration of 5.5 mmol/l was cultured for 24 hr; Control + spermine group (Control + SP) : glucose (5.5 mmol/l) +SP (10 μmol/l) DMEM complete medium treatment for 24 hr; High glucose treatment group (HG) : Complete medium with glucose concentration of 40 mmol/l was cultured for 24 hr; High glucose + spermine group (HG+SP) : glucose (40 mmol/l) + SP (10 μmol/l) was treated with DMEM complete medium for 24 hr; (5) High glucose + TβRI/II inhibitor group (HG+LY2109761) : Glucose (40 mmol/l) +LY2109761 (20 μmol/l) was completely treated with DMEM medium for 24 hr.

### Echocardiography

After 2 % isoflurane was sucked into anesthetized mice, the chest hair of mice was removed, and the cardiac function indexes of mice were detected using all-digital multi-channel beamforming, variable aperture, dynamic apodized A/D16-bit and tissue second harmonic imaging. Ejection fraction (EF), left ventricular fractional shortening (FS), left ventricular end-systolic diameter (LVIDs), and left ventricular end-diastolic diameter (LVIDd) were measured near the sternal manubrium. The heart rate and respiration of the mice were kept stable during the detection process, and the heart function of the mice was evaluated according to the animal experimental indexes of the American Society of Echocardiography.

### Masson staining

The wax blocks containing myocardial tissue were cut into 5 μm slices, dewaxed step by step with xylene, 100 %, 95 %, 80 %, and 70 % ethanol, and soaked in Zenker’s solution overnight. Rinsed with deionized water and soaked in iodine for 5 min, soaked in Na_2_S_2_O_3_ solution for 3 min, cleaned with deionized water 2-3 times, stained ponceau for 5–10 min, and then rinsed off, soaked in dodecaphosphoric molybdic acid solution and re-dyed for 5 min, aniline blue stained for 3 min, rinsed with weak acid (1 % glacial acetic acid) twice, and then soaked in weak acid for two minutes. Dehydrate with 95 %, 100 % alcohol I, and 100 % alcohol II for 2 min each time. Then, xylene I and xylene II were transparent for 2 min, and the collagen deposition of myocardial tissue was observed by an ordinary optical microscope.

### Sirius red staining

The myocardial tissue was fixed in a 10 % formalin solution, dehydrated by conventional methods, and embedded in paraffin wax. The section thickness was 3 μm, and the dewaxing method was followed (same as the Masson procedure). After staining the slices with Sirius red liquid for 1 hr, the staining liquid was washed off the surface of the slices with clean water and kept dry. The nucleus was stained with hematoxylin for 10 min, dehydrated according to the instructions, and fixed with neutral resin glue. The changes in collagen staining were observed under a common optical microscope.

### Cell migration analysis

The serum-free cell suspension of each group was added to 24-well plates. Then 500 μl DMEM containing 5 % serum was added to the corresponding hole of the 24-well plate. After culturing for 12 hr, CFs were fixed with 5 % polyformaldehyde for 30 min and stained with 0.25 % Coomassie bright blue for 5 min. The non-migrated cells in the transwell chamber were gently wiped with a cotton swab and washed with PBS three times. Observation and capture of migrating CFs in 5 fields of view with a microscope.

### ELISA

The samples contain creatine kinase isoenzyme (CK-MB), insulin, triglycerides (TG), total cholesterol (TC), troponin-I (cTnI), and lactate dehydrogenase (LDH). To conduct the assay, fix the antigen or antibody in the wells of the microplate, and use a blocking buffer to prevent nonspecific binding. The sample or standard to be tested was added to make the antigen-antibody reaction sufficient. A washing step was performed to remove unbound material and reduce background noise. Enzyme-labeled detection antibody is added, and it binds to a specific antigen or antibody. With the addition of the substrate, the enzyme-catalyzed reaction generates a measurable signal, usually a chromogenic reaction. Signal intensity was measured using a microplate reader. The concentration of antigens or antibodies in the sample was calculated from the absorbance values and the standard curve.

### EdU staining

CFs were cultured with a cell density of 1×10^6^ by adding sterile slides in a 30 mm petri dish. According to the group, the EdU test was performed 24 hr after dosing treatment. The final concentration of EdU working liquid was 10 μM (1X), and 1 ml of the above working liquid was added to the dishes of each group, and the cells were incubated in the incubator for 2 hr, washed with PBS three times, and fixed with 4 % paraformaldehyde at room temperature for 15 min. After rinsing with PBS three times, 0.3 % Triton-X-100 permeated the membrane for 15 min at room temperature. After rinsing each dish three times, 1 ml of the prepared Click reaction solution (Click Reaction Buffer 858 μl, CuSO_4_ 2 μl, Azide 488 40 μl, Click Additive Solution 100 μl) was added and incubate at room temperature for 30 min away from light. After discarding the Click reaction solution, washed three times with PBS, and used 1 ml Hoechst 33342 for nuclear staining. After rinsing three times, an anti-fluorescence quench agent was added to the slide, and the slide was sealed, observed, and photographed by a fluorescence microscope.

### Immunohistochemical analysis

According to the experimental procedure, the rat heart was removed, fixed in 4 % paraformaldehyde, and then sliced for use after conventional paraffin embedding. Dewaxing of tissue sections was all carried out in 10 mmol citrate buffer (pH 6.0), followed by high-pressure heating for 6 min. They were incubated with 3 % H_2_O_2_ for 10 min and incubated overnight with rabbit/mouse polyclonal antibodies against ODC and SSAT (dilution 1:300, Proteintech, China) at 4 °C. PBS buffer or non-immune IgG was selected as the negative control group. Subsequently, horseradish peroxide-goat anti-rabbit/mouse secondary antibody (Zhongshan Jinqiao, China) was treated at room temperature for 30 min and then developed with 3, 3-diaminobenzidine-H_2_O_2_ solution (Zhongshan Jinqiao, China). Tissue sections are stained with hematoxylin to make the nucleus appear blue under the microscope. After photographing and recording under a light mirror, the Image Pro Plus 6.0 software is applied to calculate the brown stained areas in each marked image.

### Western blot analysis

The separation gel of 12 % was prepared, and the target protein sample and Marker were added to each hole according to the group for electrophoresis (voltage 120 V, 1.5-2 hr). After film transfer (ice bath treatment, constant current 300 mA, 2 hr), the PVDF film was removed, placed in 5 % skimmed milk powder sealing solution, and sealed at room temperature for 1–1.5 hr. The PVDF membrane target protein bands were cut according to Marker position and placed in TBST liquid containing primary antibody (1:1000) at 4 °C overnight. On the second day, TBST rinsed each PVDF membrane three times for 10 min each time. TBST liquid containing secondary antibodies (1:10,000) was added and incubated at room temperature for 1.5–2 hr, followed by rinsing TBST three times, 10 min each time. Then, after the target protein PVDF membrane was immersed in the supersensitive ECL luminescent solution, the protein expression image was detected by the ultrasensitive multifunctional imager. Image J 1.46r software was used to analyze the gray value of the target protein band, and the expression level of the protein to be measured was calculated according to the internal reference expression level.

### Statistical analysis

The number of independent repetitions of all experiments reached at least three. All data were expressed as mean value ± standard error of the mean (SEM). Statistical analysis was performed by one-way ANOVA, followed by the Bonferroni multiple comparison test using GraphPad Prism (version 9.0). A value of *P*<0.05 was considered statistically significant.

## Results

### Detection of blood glucose, serum insulin, TC and TG in mice

In the 12^th^ week, blood samples from the tail vein of each group were collected to detect the blood glucose level. The contents of insulin, total cholesterol (TC), and triglyceride (TG) were detected by ELISA with blood from inner canthus veins. The results indicated that, compared to the Control group, both blood glucose levels and serum insulin concentrations were significantly higher in the T2D and T2D+SP groups (*P*<0.05). Additionally, total cholesterol (TC) and triglyceride (TG) levels were also significantly elevated in the T2D and T2D+SP groups (*P*<0.05) (Table 1).

### Detection of Serum CK-MB, cTnI, and LDH in the 12th week

In order to verify that DCM can lead to severe myocardial cell damage, the levels of serum marker enzymes (CK-MB, cTnI, and LDH) of mice in each group were detected. The results showed that compared with the Control group, the contents of serum CK-MB, cTnI, and LDH in the T2D group were significantly increased (*P*<0.05); Compared with the T2D group, the content of the above enzymes in the serum of mice in the T2D + SP group was significantly decreased (*P*<0.05) (Table 2). It can be seen that SP has protective and therapeutic effects on myocardial damage caused by HG.

### Comparison of cardiac function in each group

**Figure 1:**
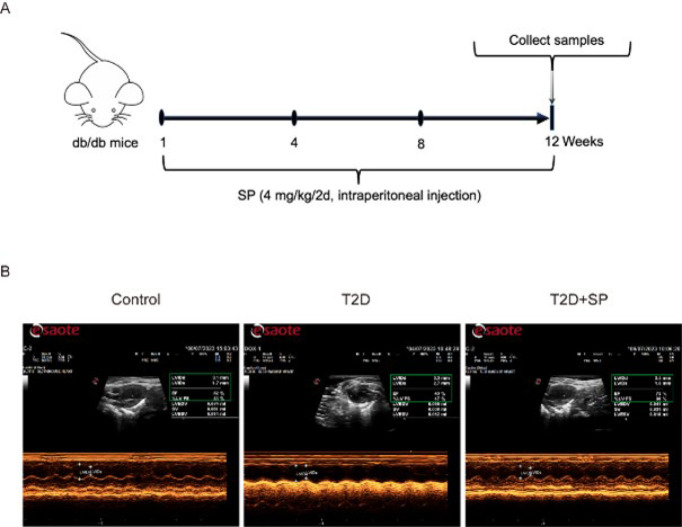
Representative imagines of echocardiograms of mice in each group at week 12

SP (4 mg/kg/2d) was injected into the mice intraperitoneally, and cardiac echocardiography was used to detect the cardiac function of each group after 12 weeks (Figure 1A). Left ventricular systolic function was assessed by observing ejection fraction (EF), left ventricular fractional shortening (FS), left ventricular end-systolic diameter (LVIDs), and left ventricular end-diastolic diameter (LVIDd). Compared with the Control group, EF and FS in the T2D group were significantly decreased, while LVIDs and LVIDd were significantly increased (*P*<0.05). Compared with the T2D group, EF, and FS in the heart of mice in the T2D+SP group were significantly increased, while LVIDs and LVIDd were significantly decreased (*P*<0.05) (Figure 1B, Table 3). The above echocardiography results suggested that at 12 weeks, T2D mice developed significant left ventricular systolic dysfunction, and exogenous spermine treatment could improve cardiac systolic function.

### Cardiac fibrosis detection of mice

At 12 weeks, each group of mice was anesthetized, their hearts were isolated and weighed, and the ratio of heart weight (HW) to tibia length (TL) was calculated. The results showed that the HW/TL ratio of mice in the T2D group was significantly increased (*P*<0.05). It was significantly decreased in the T2D+SP group (*P*<0.05) (Table 4), suggesting that the increased HW/TL ratio might be related to myocardial extracellular matrix disorder (collagen deposition). Therefore, the mouse heart morphology test was conducted to verify the results further.

**Figure 2:**
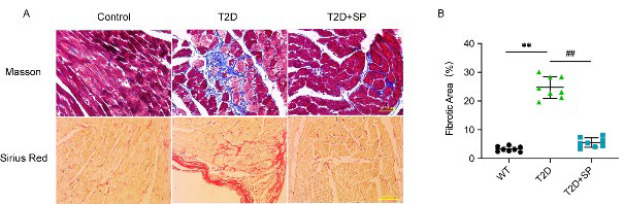
Masson and Sirius red staining of mouse myocardial tissue

Masson staining (blue area) and Sirius red staining (red area) displayed that the area of myocardial fibrosis in the T2D group was significantly increased (*P*<0.05), and more collagen deposition was observed around the degenerated and necrotic areas of myocardial tissue. Compared with the T2D group, the collagen content in the myocardial tissue of mice in the T2D+SP group was significantly decreased, and the fibrosis area was significantly decreased (*P*<0.05) (Figure 2A-B).

**Figure 3:**
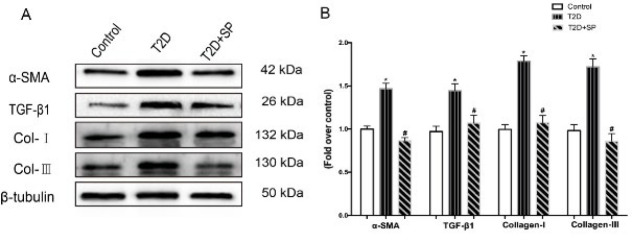
α-SMA, TGF-β1, and Collagen I/III expression was evaluated in the myocardial tissue of mice

After extracting proteins from the myocardial tissue of mice in each group, western blot analysis of related proteins showed that the expressions of α-SMA, TGF-β_1_, and Collagen I/III in the T2D group were significantly increased compared with those in the Control group (*P*<0.05). Compared with the T2D group, the above indexes in the T2D+SP group decreased significantly (Figure 3A-B).

### Detection of key enzymes in polyamine metabolism

**Figure 4:**
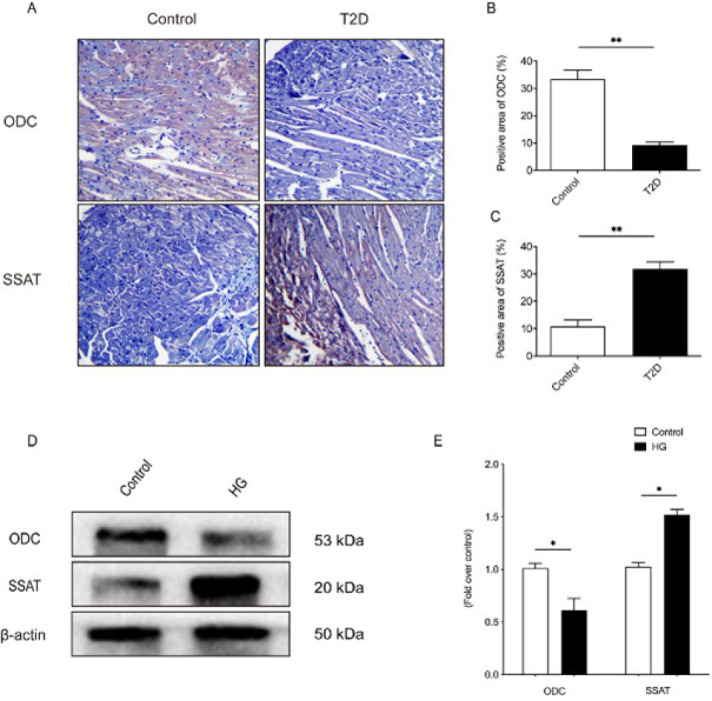
Expression of ODC and SSAT was evaluated in the myocardial tissue and CFs

In order to verify the changes in polyamine metabolism in the T2D mouse model, the expressions of ODC and SSAT, the key enzymes of polyamine synthesis, were detected by immunohistochemistry. Compared with the Control group, ODC expression in the myocardial tissue of mice in the T2D group was significantly decreased, while SSAT expression was significantly increased (*P*<0.05) (Figure 4A-C).

Since there are significant changes in polyamine metabolism in db/db mice, we infer that corresponding changes will also occur in the cell model. As anticipated, ODC expression in cardiac fibroblasts was significantly reduced in the HG group, while SSAT expression showed a significant increase (*P*<0.05) (Figure 4D-E).

### Effects of SP on proliferation and migration of myocardial fibroblasts

**Figure 5:**
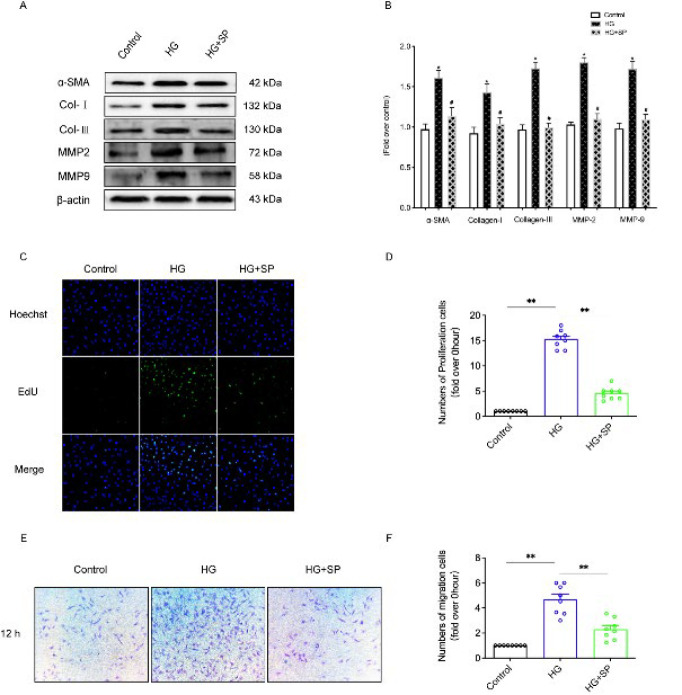
Proliferation and migration of mouse cardiac fibroblasts were detected in each group

Western blot detection of myocardial fibrosis-related proteins showed that the expressions of α-SMA, Collagen-I, Collagen-III, MMP-2, and MMP-9 in the HG group were significantly up-regulated (*P*<0.05), and the above protein expressions in HG + SP group were significantly down-regulated (*P*<0.05) (Figure 5A). It is concluded that SP can affect the phenotypic transformation of myocardial fibroblasts and the homeostasis of the extracellular matrix.

The primary mechanism of myocardial fibrosis is the excessive proliferation of myocardial fibroblasts, which leads to the synthesis and secretion of a large amount of collagen, thus affecting the balance of the extracellular matrix. In our experiment, EdU staining was used to detect the proliferation of CFs in all groups. The results showed that the proliferation ability of myocardial fibroblasts in the HG group was significantly increased (*P*<0.05). Compared with the HG group, the proliferation number of CFs in the HG + SP group was significantly reduced (*P*<0.05) (Figure 5C-D). Cell migration results confirmed that HG can promote CF migration, while SP can inhibit CF migration (Figure 5E-F).

### SP reduces the synthesis and secretion of collagen via inhibiting the TGF-β1/Smads signaling pathway

**Figure 6:**
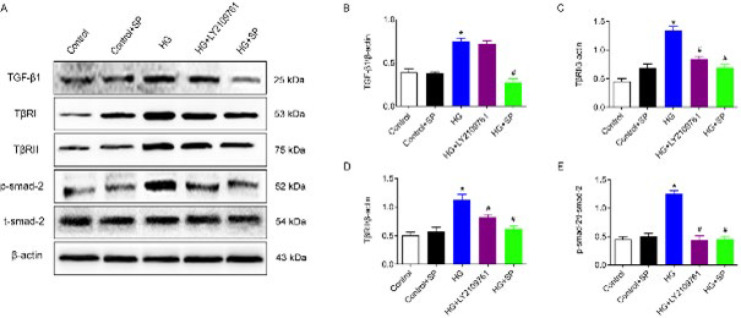
Comparison of detection of proteins related to TGF-β1/Smads pathway in cardiac fibroblasts of each group

The occurrence of myocardial fibrosis is related to the over-activation of the classical fibrotic pathway. In this study, TGF-β_1_/Smads signaling pathway inhibitor LY2109761 was added to myocardial fibroblasts treated with high glucose, and the expression of related proteins in this signaling pathway was detected by western blot (Figure 6A). The results confirmed that the expressions of TGF-β_1_, TβRI, TβRII, and p-smad-2 in cardiac fibroblasts of the HG group were significantly up-regulated. Compared to the HG group, TβRI, TβRII, and p-smad-2 expression exhibited an opposite trend in the HG + LY2109761 and HG + SP groups (*P*<0.05). In addition, SP can significantly inhibit the expression of TGF-β_1_. This suggests that SP may inhibit the activation of the TGF-β_1_/Smads signaling pathway, thereby reducing the development of myocardial fibrosis. (Figure 6B-E).

## Discussion

In recent years, the incidence of type 2 diabetes has continued to rise in China, which is closely related to the obesity epidemic(12). Studies have shown that diabetes (especially T2D) is associated with a higher risk of heart failure than high blood pressure or other types of heart disease. DCM is one of the common complications of T2D, and its destruction and influence on cardiac structure and function may be related to myocardial mitochondrial dysfunction, REDOX system imbalance, extracellular matrix (ECM) imbalance, Ca^2+^ homeostasis disruption, gene expression and post-translational modification abnormalities of related functional proteins(13, 14).

Obese Gene Receptor (OB-R), or Diabetes Gene (db), is a recessive mutant gene on chromosome 2. The leptin receptor is regulated by the obese gene (OB-R). Lepr is closely related to obesity, hypertension, diabetes, and disorders of fat metabolism (15, 16). In animal models with a db gene knockout, hyperglycemia and the activity of gluconeogenesis-related enzymes cannot be effectively controlled, even with insulin intervention. As a result, db/db homozygous mice are widely used in research on endocrine defects, nervous system disorders induced by abnormal glucose and lipid metabolism, and cardiovascular diseases.

To further investigate the pathogenesis of DCM, db gene knockout mice were used as the study subjects to observe changes in cardiac structure and function in type 2 diabetic mice. After 12 weeks of modeling, serum insulin levels, total cholesterol, triglycerides, creatine kinase-MB, cardiac troponin I, and lactate dehydrogenase were significantly higher in db/db mice than in C57BL/6 mice. Additionally, there was a marked decrease in EF and FS and a significant increase in LVIDs and LVIDd. These findings indicate that the T2D mice developed insulin resistance, myocardial tissue damage, and left ventricular diastolic and systolic dysfunction. This T2D mouse model (DCM model) successfully establishes a solid foundation for further mechanistic studies.

After myocardial fibrosis, the heart will increase in weight, which leads to thickening of the endocardium and hypertrophy of the ventricular walls, accompanied by multifocal strips of white fibers (17, 18). At the 12th week, the heart extraction of mice in each group found that HW/TL of mice in the T2D group was significantly increased, which may be related to the increase of extracellular matrix and collagen deposition in the myocardium of mice in the T2D group. The above speculation was confirmed by cardiac morphology and protein expression detection results. Masson and Sirius red staining showed that the myocardial tissue of the T2D group showed extensive and multifocal myocardial fibrosis and fibrosarcoplasmic vacuolation, especially in the subendocardial region. At the same time, there was a large amount of collagen deposition in the myocardial interstitial and degenerative necrosis area. Furthermore, we found that the protein expressions of α-SMA, TGF-β_1_, Col-I, and Col-III in the myocardial tissue of mice in the T2D group were significantly up-regulated, and the above changes could be significantly inhibited after SP treatment.

The analysis of cardiac enzymology, echocardiographic indices, and the key polyamine synthesis enzymes demonstrated that SP treatment significantly reduced CK-MB, cTnI, and LDH serum levels in the T2D group while markedly increasing the EF and FS indices. Moreover, there was a significant decrease in ODC expression and a significant increase in SSAT expression in the T2D mouse myocardial tissue, indicating a reduction in endogenous SP production. The intervention with exogenous SP notably alleviated myocardial injury and fibrosis in the T2D mice. These findings suggest that SP can improve cardiac function, protect cardiomyocytes, and mitigate myocardial fibrosis in DCM.

Spermine is a kind of polyamine that is widely distributed in organisms in the form of trivalent cations (19, 20). SP is involved in various biological processes in the body, including DNA replication, gene transcription, protein translation, post-translational modification, membrane potential maintenance, and ion channel switching(21, 22). Cell migration results in the *in vitro* experiment confirmed that SP can inhibit CF migration. In addition, endogenous SP has protective effects on the heart and nervous system, which can alleviate cardiac aging by improving mitochondrial biological function (7).

The expression of key proteins in the extracellular matrix of myocardial fibroblasts was analyzed, revealing that HG increased the expression of specific marker proteins such as α-SMA, MMP-2 (which degrades collagen-IV), MMP-9 (which degrades laminin and fibronectin), as well as collagen-I and III. This indicates that high glucose can activate CFs and promote myocardial fibrosis. However, the elevated expression of MMP-2 and MMP-9 can lead to excessive extracellular matrix degradation, expanding the intercellular space and facilitating the secretion of more collagen I and III by CFs. Following SP intervention, the expression of these proteins was significantly down-regulated, suggesting that exogenous SP can inhibit CF activation and collagen secretion. The proliferation of CFs is another key factor in myocardial fibrosis, and EdU staining confirmed that high glucose stimulation promotes CF proliferation, while SP significantly reduces these effects. Further investigation showed that high glucose markedly reduced ODC expression and increased SSAT expression in CFs, consistent with the animal experiment results. These findings indicate that the development of diabetic myocardial fibrosis is linked to an imbalance of polyamines in the body.

Transforming growth factor-β_1_, a member of the TGF-β superfamily is a secreted protein that can exist both extracellular and intracellular. It plays an important role in promoting the transformation of myocardial fibroblasts into myofibroblasts, transmitting biological signals to the nucleus to activate collagen I and collagen III gene expression, regulating the MMP/TIMP system, and affecting ECM homeostasis (23, 24). In TGF-β_1_/Smads signaling pathway, the downstream signaling protein of TGF-β_1_ is the Smads family, which is divided into three types according to function: receptor type (R-Smads: Smad2/3), universal type (Co-Smads: Smad4) and inhibited type (I-Smads: I-SMADS) (25-27). After binding with TβRII, the extracellular TGF-β_1_ molecule activates regulatory enzymes, which phosphorylates Smad2 and Smad3 and connects with Smad4 to form the Smad complex, which enters the nucleus and regulates the expression of target genes under the action of transcription factors (28, 29).

To elucidate the relationship between SP and the TGF-β_1_/Smads signaling pathway in the development of myocardial fibrosis of DCM, in this study, SP and an inhibitor of this signaling pathway (LY2109761) were added to HG-treated CFs. The results showed that high glucose significantly up-regulated the expression of TGF-β_1_, TβRI, TβRII, and Smad2 in CFs. However, both SP and LY2109761 significantly down-regulated the expression of these proteins, although LY2109761 had little effect on TGF-β_1_ expression, likely due to its role as a dual inhibitor of TβRI/II. These findings suggest that SP alleviates myocardial fibrosis in DCM by inhibiting the activation of the TGF-β_1_/Smads signaling pathway and reducing the proliferation, differentiation, and collagen secretion of CFs.

**Table 1:** Detection of blood glucose, serum insulin, TC, and TG levels in mice from each group at the 12th week (n=8)

Group	Glucose (mmol/l)	Insulin (ng/ml)	Total cholesterol (mmol/l)	Triglyceride (mmol/l)
Control	5.51±0.57	0.23±0.05	2.44±0.41	1.23±0.16
T2D	30.35±3.64^*^	0.55±0.05^*^	5.76±0.34^*^	2.82±0.35^*^
T2D+SP	28.93±2.62^*^	0.46±0.03^*^	5.54±0.52^*^	2.64±0.21^*^

*P<0.05 vs Control group. T2D: type 2 diabetes; SP: spermine; TC: total cholesterol ; TG: triglyceride

**Table 2:** Detection of serum marker enzymes CK-MB, cTnI, and LDH levels in each group of mice (n=8)

Group	CK-MB (U/l)	cTnI (pg/ml)	LDH(U/l)
Control	60.22±6.33	10.19±1.33	184.12±17.87
T2D	149.71±14.65^*^	34.44±3.87^*^	558.06±50.12^*^
T2D+SP	84.58±8.99^#^	25.71±2.32^#^	254.91±24.59^#^

*P<0.05 vs Control group;#*P*<0.05 vs T2D group. T2D: type 2 diabetes; SP: spermine

(A) Establishment of type 2 diabetic mouse model. (B) Echocardiographic indicators were assessed for mice in each group. SP: spermine; T2D: type 2 diabetes

**Table 3:** Comparison of cardiac echocardiographic results of mice in each group at week 12 (n=8)

Group	EF (%)	FS (%)	LVIDs (mm)	LVIDd (mm)
Control	80.23±6.68	42.54±2.34	1.18±0.14	2.13±0.25
T2D	50.87±6.39^*^	21.43±3.46^*^	3.42±0.39^*^	4.41±0.39^*^
T2D+SP	71.67±7.87^#^	34.56±4.11^#^	1.26±0.09^#^	1.95±0.23^#^

EF: ejection fraction; FS: left ventricular fractional shortening; LVIDs: left ventricular internal dimension systole; LVIDd: left ventricular internal dimension diastole; T2D: type 2 diabetes; SP: spermine. *P<0.05 vs Control group; #*P*<0.05 vs T2D group

**Table 4:** Ratio of heart weight (HW) and tibia length (TL) of mice was measured after 12 weeks in each group (n=8)

Group	HW/TL（mg/mm）
Control	6.12±0.65
T2D	8.81±0.87^*^
T2D+SP	6.32±0.59^#^

*P<0.05 vs Control group; #*P*<0.05 vs T2D group. T2D: type 2 diabetes; SP: spermine

(A) Representative images of Masson staining (blue region) and Sirius red staining (red region) of whole mice myocardial tissue in Control, T2D, and T2D+SP (n = 8). (B) Fibrosis area of each group was determined (n = 8). **P<0.01 vs Control group; ##*P*<0.01 vs T2D group (n = 8). T2D: type 2 diabetes; SP: spermine

(A) Protein expression levels of α-SMA, TGF-β1, and Collagen-I/III were examined by western blot (n = 3). (B) Statistical analysis of α-SMA, TGF-β1, and Collagen-I/III. *P<0.05 vs Control group; #*P*<0.05 vs T2D group (n = 3). α-SMA: alpha-smooth muscle actin; TGF: transforming growth factor

(A) Representative immunohistochemistry staining showed the expression and quantitative analysis of ODC and SSAT, the key enzymes of polyamine synthesis. (B) The positive area of ODC was significantly reduced in T2D compared with Control. (C) The positive area of SSAT was increased significantly in T2D compared with Control. (D) A representative western blot showing the protein levels of ODC and SSAT in CFs, with the protein levels normalized by β-actin (n = 3). (E) Statistical analysis of ODC and SSAT. *P<0.05 vs Control group; **P< 0.01 vs Control group (n = 2). ODC: ornithine decarboxylase; SSAT: SPD/spermine N1-acetyltransferase; CFs: cardiac fibroblasts; T2D: type 2 diabetes

(A) Protein expression levels of α-SMA, Collagen-I/III, and MMP-2/9 were examined by western blot (n = 3). (B) Statistical analysis of α-SMA, Collagen-I/III, and MMP-2/9. *P<0.05 vs Control group; #*P*<0.05 vs HG group (n = 3). (C) EdU staining was used to detect the effect of SP on the proliferation of myocardial fibroblasts (n = 3). (D) Statistical analysis of the effect of SP on the numbers of proliferation cells of myocardial fibroblast. (E) The Transwell assay is used to detect CF migration. (F) Statistical analysis of the number of migrating cells of cardiac fibroblasts. *P<0.05 vs Control group; #*P*<0.05 vs HG group (n = 3). α-SMA: alpha-smooth muscle actin; MMP: matrix metalloproteinase; HG: hyperglycemia; EdU: 5-ethynyl-2’-deoxyuridine; SP: spermine; CFs: cardiac fibroblasts

(A) Protein expression levels of TGF-β1, TβRI/ II, p-smad-2, and t-smad-2 were examined by western blotting (n = 3). (B-D) Statistical analysis of TGF-β1, TβRI/II, and p-smad-2. *P<0.05 vs Control group; #*P*<0.05 vs HG group (n = 3). TGF: transforming growth factor

## Conclusion

Under diabetic (hyperglycemic) conditions, myocardial fibroblasts become overactivated, proliferate excessively, and secrete collagen. SP can inhibit the activation of the TGF-β_1_/Smads signaling pathway and down-regulate the expression of collagen I and III genes, thereby reducing collagen synthesis in CFs. This study sheds light on SP’s role and molecular mechanism in myocardial fibrosis associated with DCM from a novel perspective, offering new insights into the prevention and treatment of DCM.

## Declaration

We have not used any AI tools or technologies to prepare this manuscript.
